# Supplementary material for: Virtual Active Learning to Maximize Knowledge Acquisition in Nursing Students: A Comparative Study
Source: Nurs Rep. 2024 Jan 8;14(1):128–39. doi: 10.3390/nursrep14010011 (PMC10801574; doi:10.3390/nursrep14010011)
Supplement: Supplementary file 1 [file nursrep-14-00011-s001.zip › nursrep-2717423-supplementary.pdf]

Supplementary Table S1. Contents taught in both groups

List of topics
1. The Behavioral Theory
2. Types of conditioning
3. Classical conditioning
4. Operant conditioning
5. Behavior modification tools
